# Underload on the Road: Measuring Vigilance Decrements During Partially Automated Driving

**DOI:** 10.3389/fpsyg.2021.631364

**Published:** 2021-04-15

**Authors:** Thomas McWilliams, Nathan Ward

**Affiliations:** Department of Psychology, Tufts University, Medford, MA, United States

**Keywords:** vigilance, underload, passive fatigue, mind-wandering, partial automation, driving simulation

## Abstract

Partially automated vehicle technology is increasingly common on-road. While this technology can provide safety benefits to drivers, it also introduces new concerns about driver attention. In particular, during partially automated driving (PAD), drivers are expected to stay vigilant so they can readily respond to important events in their environment. However, using partially automated vehicles on the highway places drivers in monotonous situations and requires them to do very little. This can place the driver in a state of cognitive underload in which they experience a very small amount of cognitive demand. In this situation, drivers can exhibit vigilance decrements which impact their ability to respond to on-road threats. This is of particular concern in situations when the partially automated vehicle fails to respond to a potentially critical situation and leaves all responsibility to safely navigate to the driver. This paper reviews situations that lead to vigilance decrements and characterizes the different methodologies of measuring driver vigilance during PAD, highlighting their advantages and limitations. Based on our reading of the literature, we summarize several factors future research on vigilance decrements in PAD should consider.

## Introduction

Vehicle automation offers promising benefits to drivers and can describe a number of different vehicle technologies despite potential confusion about exact definitions of “self-driving cars.” To help with classifying vehicle automation, SAE International has defined 6 levels of vehicle automation ranging from no automation to full automation (Table 1; SAE International, 2014). Our use of the term partially automated driving (PAD) refers to SAE levels 1, 2, or 3. Level 1 automation (Driver Assistance) includes drivers assistance systems such as Adaptive Cruse Control (ACC) or Lane Keeping Assist (LKA). ACC, like traditional cruise control, allows the vehicle to maintain a speed without the driver’s input but unlike traditional cruise control can also change the speed of the vehicle depending on the speed of surrounding vehicles. LKA monitors the lane markings on the road and helps steer the vehicle to keep it within the lane. Level 1 automation allows for only one of these systems to be active at a time. Level 2 (Partial Automation) allows for ACC and LKA to work at the same time. Level 3 (Conditional Automation) means the automated system can perform all aspects of the dynamic driving task which includes even more advanced versions of ACC and LKA along with other automated systems. However, during PAD drivers must monitor the vehicle and intervene in any safety critical event to which the automation does not properly respond. The common characteristic between these levels of automation is that the system can perform at least some of the dynamic driving tasks, but drivers are required as a fallback if the automation encounters problems. For example, if lane markings are unclear and the automation can no longer keep the vehicle in an appropriate position on the road, then drivers must be ready to step in and take control of the vehicle to ensure safe transportation for themselves, passengers, and other drivers.

**TABLE 1:** Summary of the SAE levels of automation (SAE International, 2014).

**Sae Level**	**Name**	**Execution of Dynamic Driving Task**	**Monitors Driving Environment**	**Fallback**
0	No Automation	Human Driver	Human Driver	Human Driver
1	Driver Assistance	Human Driver and System	Human Driver	Human Driver
2	Partial Automation	System	Human Driver	Human Driver
3	Conditional Automation	System	System	Human Driver
4	High Automation	System	System	System
5	Full Automation	System	System	System

*This review focuses on levels 1, 2, and 3 which allow for the system to be responsible for the execution of part or all of the dynamic driving task but requires a human driver as a fallback.*

Partially automated vehicles offer promising benefits to drivers, however, there are growing safety concerns as the prevalence of these vehicles increases on-road. For example, during PAD drivers must supervise the automation and be ready to intervene at any moment. In other words, PAD requires drivers to do little more than supervise the automation for extended periods of time. Indeed, a recent analysis of an on-road higher-level partially automated vehicle found that driver interactions with the vehicle were only required every 150 to 250 miles of driving (Corcoran et al., 2019). This means that drivers may spend extended periods of time not engaging with the vehicle. This sustained lack of interaction may cause a state of cognitive underload and in turn lead to vigilance decrements.

### Vigilance Decrements and Cognitive Underload

Vigilance, sometimes referred to as sustained attention, has been studied in depth since Mackworth’s research on radar operators and the famous “Mackworth’s Clock” experiments in the 1940’s and 50’s (Mackworth, 1948, 1951). One important discovery at this time was that performance on vigilance tasks (i.e., tasks in which infrequent targets are tracked over time) declines over time (Mackworth, 1948). Declines in performance primarily take place over the first 15 min of the task, and performance levels off over the next 30 to 90 min (Levine et al., 1973; Teichner, 1974; Parasuraman, 1979). This decline is the vigilance decrement, where people are less likely to detect and properly respond to an infrequent target stimulus (See et al., 1995).

In transportation, multiple factors influence whether or not drivers are in a situation prone to causing a vigilance decrement. One important factor is time spent on the task. A short task may not show any decrement in vigilance, but longer sustained tasks are more likely to show vigilance decrements (Levine et al., 1973; Teichner, 1974; Parasuraman, 1979). Another important factor is task demand; tasks that place little demand on the performer are more likely to lead to vigilance decrements (Thomson et al., 2015; Danckert and Merrifield, 2018). Vigilance decrements also depend on how often someone engages with the task. When target stimuli are rare and the task is performed infrequently, then vigilance decrements are likely to occur (Parasuraman and Davies, 1977). It is easy to draw a parallel between a scenario that will cause a vigilance decrement and a scenario of PAD. When driving a partially automated vehicle it is common to spend extended periods of time monitoring the partially automated vehicle. This monitoring task places little demand on the driver, and driver interventions with the vehicle are infrequent when automation is operating properly. The driver experiences a vigilance decrement during PAD because scenarios of monotonous PAD cause a state of cognitive underload in the driver (Körber et al., 2015a; Cabrall et al., 2016; Solís-Marcos et al., 2017; Cunningham and Regan, 2018).

A state of cognitive underload is especially common during continuous, monotonous, and low demand driving scenarios (Ma et al., 2018). Essentially this means that the driver is performing such a low demand task, and is experiencing so little arousal, that they disengage from the task and their task performance actually suffers because of it. This is the vigilance decrement which arises from a state of cognitive underload, which inhibits their ability to supervise and intervene with the automation in an emergency (Saxby et al., 2013). For example, if the partially automated vehicle does not detect an object in the forward roadway, then the driver would need to intervene to avoid a collision. If driver vigilance has declined due to cognitive underload, then their reaction may be slowed, making it difficult to avoid a collision.

**FIGURE 1:**
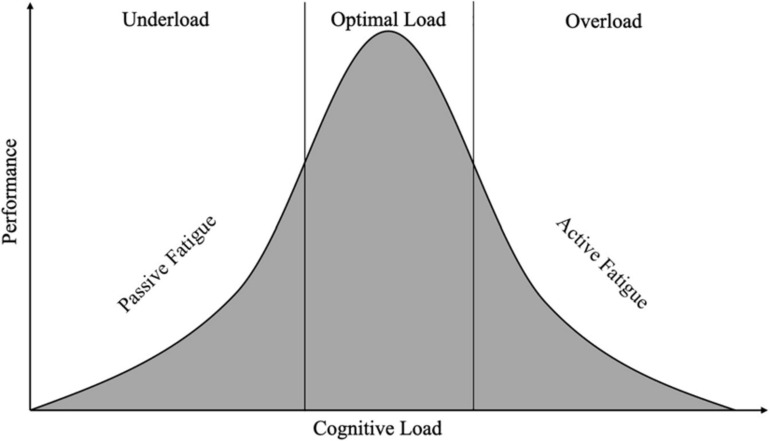
Curve demonstrating the relationship between cognitive load and task performance. The left section shows when someone is underloaded and experiencing passive fatigue which is associated with poor task performance. The center section shows how a certain amount of cognitive load is associated with better performance. The right section shows overload which leads to active fatigue and is associated with poor task performance.

There are several potential underlying causes behind cognitive underload leading to vigilance decrements in driving, although much of the research to date has been focused on different types of manual driving. Passive fatigue is the depletion of attentional resources over time due to a low demand task (Desmond and Hancock, 2001; Saxby et al., 2013; Körber et al., 2015a). Passive fatigue differs from active fatigue, which depletes attentional resources due to high task demands, whereas passive fatigue depletes attentional resource due to low task demands. This can be visualized by thinking of an inverted U similar to the Yerkes-Dodson curve (Yerkes and Dodson, 1908). Driving research often focuses on the right half of the curve and is concerned with active fatigue causing performance decrements (when drivers are overloaded). Cognitive underload and passive fatigue concern the left half of the plot which is also capable of causing performance decrements (Figure 1). Essentially what happens is that the goal of maintaining attention on a low demand task still requires and depletes attentional resources (Thomson et al., 2015), which can lead to performance changes characterized as vigilance decrements.

In addition to passive fatigue, vigilance decrements may be a result of mind-wandering (Smallwood and Schooler, 2006). After performing a monotonous task for an extended period of time, attention may be withdrawn from the primary task and a new internal focus of attention is generated, perhaps in part to ward off boredom (Malkovsky et al., 2012; Shepherd, 2019). This alternate focus of attention can consist of Task Unrelated Thoughts (TUTs) (Smallwood, 2013), which in the case of PAD would be thoughts unrelated to driving and/or monitoring the partially automated vehicle. Mind-wandering is an umbrella term that refers to TUTs and a broad range of other intentional and unintentional ways of thinking that are independent of a primary task or stimulus of interest (Seli et al., 2018). Irrespective to their specific content, these self-generated thoughts reallocate attentional resources away from the primary external task, leading to failures in performance characteristic of vigilance decrements (Smallwood and Schooler, 2006).

Regardless of the underlying causes of vigilance decrements, it is becoming increasingly clear that PAD is going to have an impact on driver vigilance, which could have severe implications for transportation safety at least until we get to levels of full automation (i.e., drivers are no longer required to monitor partially automated systems that require infrequent interactions). One way to increase transportation safety in PAD contexts is to have countermeasures to vigilance decrements that may occur when drivers are tasked with monitoring partially automated systems. In order to generate countermeasures to vigilance decrements in PAD contexts, we must first characterize how vigilance is measured in PAD, which is the goal of this narrative review.

### PAD Compared to Manual Driving

Research on partially automated vehicles and driver attention primarily takes place in either real-world or simulated highway driving scenarios. Highway environments are where most forms of partially automated vehicles are designed to work. These highway environments are monotonous; there is low to moderate traffic, the background scenery is limited, the weather is relatively clear, and there is an infrequent need for drivers to respond to their surrounding environment. These driving environments place little demand on drivers, and adding partially automated features to the vehicle means that drivers have even less to engage with during the drive. In comparison to PAD, researchers can also monitor drivers during manual driving. Manual driving, or SAE automation level 0, means drivers are in full control of the vehicle at all times, although this does not preclude certain non-automation-related technologies, such as automatic transmissions or anti-lock braking systems (SAE International, 2014). In general, manual driving is more engaging than PAD and is less likely to lead to cognitive underload, which is why many of the studies included in this review use manual driving as a benchmark for comparing vigilance in PAD contexts (Saxby et al., 2008, 2013; Neubauer et al., 2012; Greenlee et al., 2018; Heikoop et al., 2018).

### Additional Constraints for Literature Review

Since PAD means the automated system takes the responsibility for controlling the vehicle away from the driver most of the time, the driver is less engaged with the vehicle. One potential confound in this situation is the increase in secondary task distractions. Indeed, during PAD, drivers are more likely to use a smartphone or engage in other non-driving activities behind the wheel (Wandtner et al., 2018). Designers of commercial partially automated vehicles are aware of this; therefore, drivers are instructed to avoid tasks they would not perform when driving a non-partially automated vehicle. In addition, laws regarding cellphone use and other distractions are still in effect when engaging in PAD, though this might not dissuade drivers from multitasking. While non-driving tasks during PAD are a valid concern, especially as they may lead to situations of active fatigue or overload, the primary focus of the current review is on situations in which drivers are using partially automated technology as it was designed to be used. In other words, we focus on situations in which drivers have the primary goal of monitoring the automation and the driving environment at all times without secondary tasks. For these reasons, we narrowed our scope to studies that did not require the performance of secondary tasks. Furthermore, we excluded studies that allowed participants to voluntarily perform secondary tasks. We also tried to avoid studies focusing on vigilance decrements in PAD related to driver sleepiness and sleep related fatigue rather than underload (Wierwille et al., 1994; Merat et al., 2012; Heikoop et al., 2017; Jarosch et al., 2019).

### Measuring Vigilance in PAD

Based on our reading of the extant literature, there are two ways to classify tools used to measure vigilance during PAD; offline measures and online measures. Offline measures can assess the general cognitive state of the driver over a period of time. These measures are usually based on self-report data. Offline measures are collected *before and/or after* periods of driving. For example, after completing a drive, drivers may provide self-reports indicating possible vigilance decrements. These offline measures are unable to indicate a driver’s state in real-time, although they are usually non-intrusive and easy to implement.

On the other hand, online measures use variables that correlate to active driver states and can be collected *during* periods of PAD. For example, an online measure can monitor how quickly a driver can avoid an eminent collision, which is just one online metric of vigilance performance. These online measures have a much higher temporal sensitivity than offline measures but can also be somewhat intrusive and harder to implement both in terms of logistics and cost. To help track how different measures are combined Table 2 list all the papers we review, this table is primarily organized by offline and online measures. In the next sections, we will characterize the specific types of offline and online measures currently used to study vigilance decrements in PAD.

**TABLE 2:** Table of papers that investigate driver vigilance during PAD.

**No.**	**Authors**	**Offline Measures**	**Online Measures**	**Apparatus**	**Drive Length (Min)**	**Automation**	***N***	**Design**	**Discussed Cause**
1	Biondi et al., 2018	Driver Engagement Questionnaires	Detection Task, HR	On-Road	60	Manual, PAD	22	W	Both
2	Cisler et al., 2019		Critical Events (10), Detection Task, EEG, Eye-Tracking, HR, Questionnaire Probes	SimL	10	PAD	25	W	Both
3	Funkhouser and Drews, 2016		Critical Event (1), HR	SimM	2, 5, 10	PAD	24	W	
4	Gaspar and Carney, 2019		Eye-Tracking	On-Road	∼13	Manual, PAD	10	W	
5	Greenlee et al., 2018	Driver Engagement Questionnaires, NASA-TLX	Critical Events (15)	SimM	40	PAD	22	W	
6	Heikoop et al., 2017	Driver Engagement Questionnaires, NASA-TLX	Detection Task, Eye-Tracking, HR	SimH	40	PAD	22	W	
7	Heikoop et al., 2018	Driver Engagement Questionnaires, NASA-TLX	Eye-Tracking, HR, Questionnaire Probes	SimH	40	PAD	33	W	
8	Jarosch et al., 2019		Critical Event (1), Eye-Tracking	SimH	50	PAD	66	W	PF
9	Körber et al., 2015a		Detection Task, Eye Tracking, Questionnaire Probes	SimH	42.5	PAD	20	W	Both
10	Körber et al., 2015b		Critical Event (1)	SimM	24	PAD	23	W	Both
11	Louw and Merat, 2017		Critical Events (2), Eye-Tracking	SimH	20	Manual, PAD	60	M	MW
12	Louw et al., 2015		Critical Events (2), Eye-Tracking	SimH	20	Manual, PAD	30	M	Both
13	Merat et al., 2012		Critical Event (1), Eye-Tracking	SimH	45	Manual, PAD	50	W	
14	Merat et al., 2014		Critical Event (1), Eye-Tracking	SimH	∼45	Manual, PAD	37	W	
15	Neubauer et al., 2012	Driver Engagement Questionnaires, NASA-TLX	Critical Event (1)	SimM	35	Manual, PAD	184	B	PF
16	Saxby et al., 2008	Driver Engagement Questionnaires, NASA-TLX	Critical Event (1)	SimL	10, 30	Manual, PAD	168	B	PF
17	Saxby et al., 2013; Study 1	Driver Engagement Questionnaires, NASA-TLX		SimM	10, 30, 50	Manual, PAD	108	B	PF
17	Saxby et al., 2013; Study 2	Driver Engagement Questionnaires, NASA-TLX	Critical Event (1)	SimM	10, 30	Manual, PAD	168	B	PF
18	Shen and Neyens, 2014		Critical Event (1)	SimM	15	LKA, PAD	36	M	
19	Solís-Marcos et al., 2017	NASA-TLX	Detection Task, EEG	SimM	5	Manual, PAD	20	W	PF
20	Zeeb et al., 2015		Critical Event (3), Eye-Tracking	SimH	28	PAD	89	W	

*Parentheses after critical events indicates the number of critical events used per drive. On-Road = real-world highway driving, SimL = low fidelity driving simulator, SimM = medium fidelity driving simulator, SimH = high fidelity driving simulator (see McWilliams et al., 2018 for details). ^∼^estimate of drive length when not directly reported. W = within-subject design, B = between-subject design, M = mixed-effect design. The Discussed Cause column indicates if either passive fatigue (PF), mind-wandering (MW), or both (Both) were discussed as possible causes to observed vigilance decrements.*

## Offline Measures of Vigilance in PAD

As mentioned above, offline measures typically involve some type of self-report, which can take a variety of different forms in terms of the specific types of questionnaires used as offline measures to monitor driver vigilance. Some of these are intended to measure driver workload, whereas others are intended to measure driver engagement. Since offline measures are not collected during the drive they are unable to show fluctuations in driver states. Also, these measures rely on self-reports after the drive so they are subject to response biases and the participant’s ability to recall their state during the drive. However, offline measures can be included in almost any experimental design since they are not completed during the driving portion of the experiment and require minimal equipment and time to implement. Offline measure can also provide insight into subjective driver experiences which might not be measurable otherwise.

### Driver Workload Questionnaires

One of the most commonly used driving workload questionnaires in PAD research is the NASA Task Load Index (NASA-TLX), which consists of a 21-point scale for several dimensions of physical and cognitive workload (Hart and Staveland, 1988; Hart, 2006). The NASA-TLX measures mental demand, physical demand, temporal demand, effort, performance, and frustration and generates an overall workload score from the numerical responses to these dimensions. Participants often fill out this scale after a period of PAD or manual driving (Neubauer et al., 2012; Heikoop et al., 2017, 2018; Greenlee et al., 2018). After a period of PAD, drivers often report lower workload scores on the NASA-TLX (Saxby et al., 2008, 2013; Solís-Marcos et al., 2017). For example, Saxby et al. (2008) found that after PAD, participants reported significantly lower levels of workload on the NASA-TLX than they did after a manual driving condition. In general, response scores on the NASA-TLX are lower after periods of cognitive underload when vigilance decrements are present (Saxby et al., 2008; Solís-Marcos et al., 2017).

The NASA-TLX was not initially developed to specifically measure vigilance but instead to measure a more general workload experience when performing a task (Hart, 2006), and it only reflects workload after a possible vigilance decrement has occurred. This must be kept in mind when interpreting NASA-TLX scores in PAD studies focused on vigilance decrements. Non-etheless, the NASA-TLX scores provide insight into the workload participants experience, with lower scores being more common during drives designed to induce underload and higher scores being more common during drives designed to induce overload (Saxby et al., 2008). Furthermore, the NASA-TLX is quick to administer and requires minimal equipment or financial burden.

### Driver Engagement Questionnaires

In addition to the NASA-TLX, we found two common questionnaires used to measure vigilance decrements in PAD contexts. The Dundee Stress State Questionnaire (DSSQ) and Short Stress State Questionnaire (SSSQ) (which is derived from the DSSQ) contain questions pertaining to engagement, worry, and distress (Matthews et al., 2002; Helton, 2004). DSSQ is also sometimes used as a retrospective mind-wandering measurement (Smallwood et al., 2006; Riby et al., 2008; Maillet and Rajah, 2013) perhaps in part because the engagement subsection includes items such as “my mind is wandering very much” (Matthews et al., 2002) and “I was committed to attaining my performance goals” (Helton, 2004). In addition, these questionnaires are typically administered both before and after periods of PAD to allow researchers to focus on relative changes in driver engagement. For example, when a PAD scenario induces cognitive underload, drivers often report lower levels of engagement after the drive compared to before the drive (Neubauer et al., 2012; Saxby et al., 2013; Heikoop et al., 2017, 2018; Greenlee et al., 2018). In one study, Neubauer et al. (2012) had participants drive without any automation or drive with the option of using partial automation. Pre-drive measures of engagement were overall higher than post-drive measures, however, engagement ratings decreased significantly more when drivers used automation. This suggests that one characteristic of underload during PAD is drivers are less engaged with the driving environment. Relatedly, drivers also report higher levels of mind-wandering after PAD compared to after manual driving (Körber et al., 2015a), which further suggests that drivers are less engaged with the driving environment during PAD.

Similar to the NASA-TLX, the DSSQ and SSSQ were not initially designed to specifically measure vigilance decrements so this must be considered when interpreting results. Regardless, lower engagement scores are found after periods of PAD so these engagement scores can help identify potential vigilance decrements after they occur (Neubauer et al., 2012; Saxby et al., 2013; Heikoop et al., 2017, 2018; Greenlee et al., 2018). Different from the NASA-TLX, in PAD contexts focused on vigilance decrements, the DSSQ and SSSQ are frequently presented prior to a scenario, as well as after the scenario. This could be problematic in that participants may be more susceptible to biases or more aware of their own mental state during the experiment. In other words, administering driver engagement surveys *prior* to PAD may impact driver engagement *during* PAD. On the other hand, and similar to the NASA-TLX, these two questionnaires are easy to implement and require minimal equipment or financial burden.

## Online Measures of Vigilance in PAD

Online measures typically require participants to respond to stimuli or involve monitoring psychophysiological changes in drivers during PAD. Since online measures are collected during the drive, they are able to show time sensitive changes in driver states. These measures may be cumbersome to participants because they have to perform additional tasks or may be invasive because they require participants to wear various physiological sensors that are susceptible to signal noise resulting in data loss. However, online measures can show gradual changes in vigilance decrements better than offline measures can. Also, online measures have the potential to detect vigilance decrements as they occur, which is important for future research if we hope to use interventions to mitigate vigilance decrements in real-time.

### Safety Critical Events

Some of the most commonly used online measures to monitor vigilance decrements during PAD are safety critical events. These often consist of automation failures when the vehicle’s partially automated system disengages on its own, the vehicle fails to detect an object blocking the roadway, or the vehicle requests drivers to take full control (Saxby et al., 2008, 2013; Merat et al., 2012, 2014; Neubauer et al., 2012; Shen and Neyens, 2014; Körber et al., 2015b; Louw et al., 2015; Zeeb et al., 2015; Funkhouser and Drews, 2016; Louw and Merat, 2017; Greenlee et al., 2018; Cisler et al., 2019; Jarosch et al., 2019). In PAD, vigilance decrements are commonly characterized by drivers responding slower to safety critical events the longer they are driving and also compared to non-PAD. Response times to how quickly the driver presses the brake pedal, swerves out of the way, or takes another action to avoid the collision are some of the most common ways to measure safety critical events in PAD compared to manual driving. For example, Saxby et al. (2013) had participants complete a PAD scenario during which a parked van suddenly pulled out in front of their vehicle. Drivers had to take control of the vehicle to avoid a collision. Responses to this safety critical event were slower during PAD compared to manual driving, which the authors used as an indicator of vigilance decrements (Saxby et al., 2013). In addition to comparing PAD to manual driving, responses to safety critical events also tend to slow over the course of an entirely PAD scenario (Funkhouser and Drews, 2016; Greenlee et al., 2018; Cisler et al., 2019), which is consistent with the idea that vigilance declines over time.

To reiterate, compared to PAD contexts, vigilance decrements are smaller during manual driving indicated by faster responses to safety critical events during manual driving (Saxby et al., 2008, 2013; Neubauer et al., 2012). Interestingly, vigilance decrements can also differ between levels of automation in PAD. For example, Shen and Neyens (2014) had participants drive on a simulated highway and found faster response times to critical events when using SAE level 1 automation compared to level 2 automation. This suggests higher levels of automation lead to larger vigilance decrements in part because higher levels of automation lead to even less engagement with the vehicle, which exacerbates any vigilance decrement.

Response times to safety critical events are a very clear way to monitor and see the consequences of vigilance decrements during PAD because safety critical events are designed to mirror the types of situations that are of concern when vigilance decrements occur. Furthermore, for driving simulator research, safety critical events are quite easy to implement. Despite being one of the most commonly used online measures of vigilance decrements in PAD, safety critical events have some limitations. For example, when using safety critical events, it is important to be aware of how scenarios are designed in terms of the frequency and time between critical events. If events are too frequent and close together, then drivers may actually stay in a state of high vigilance (Parasuraman, 1979). On the flip side, if only a few safety critical events are included, the statistical analysis may be problematic due to lower power. For example, Louw et al. (2015) used only two safety critical events in each drive and found no significant difference in response between PAD and manual driving across 30 participants. In addition, safety critical events in PAD may lead to collisions. Much of this research is conducted in driving simulators so there is no physical risk to participants, but crashing may still impact a participant’s psychological state to a degree that driving scenarios are typically stopped after a crash (Caird and Horrey, 2011), which means that the length of drives across participants may be inconsistent. Along with this, safety critical events can only be safely used in driving simulations and are less applicable in on-road settings.

### Detection Tasks

A variety of different styles of detection tasks are used to actively monitor vigilance decrements during PAD, and many of these tasks are reminiscent of more traditional vigilance tasks done outside of driving research. Detection tasks require drivers to monitor and respond to an intermittent stimulus. When vigilance is high, responses are fast and accurate, but as vigilance declines, responses slow and become less accurate. One popular detection task paradigm in driving research is the Detection Response Task or DRT (International Organization for Standardization(ISO), 2016). The DRT usually consists of a response button affixed to the driver’s finger and different stimuli (e.g., light, tone, or vibration). Whenever the stimulus is presented and detected, either a flash of an LED, an auditory tone, or a vibrotactile motor, drivers are instructed to respond by pressing the response button as quickly as possible. The DRT can be easily implemented in driving simulators and is also used in on-road settings during PAD. For example, Biondi et al. (2018) used a vibrotactile version of the DRT in a real-world PAD context. Specifically, while driving on a highway, drivers had slower DRT response times to the vibrotactile stimuli during PAD compared to manual driving. In addition, DRT responses were even slower during the second half of the PAD session (Biondi et al., 2018). This shows the impacts of a vigilance decrement on the task performance compared to manual driving, as well as how vigilance declines over time even within PAD scenarios.

In addition to the single stimulus detection tasks, PAD researchers have used a multi-stimulus detection tasks sometimes referred to as oddball tasks (Körber et al., 2015a; Solís-Marcos et al., 2017). These oddball tasks often have two different stimuli (e.g., a high frequency tone and a low frequency tone), and drivers are required to only respond to one of the two stimuli with a button press. In contrast to single stimulus detection tasks, response times in multi-stimulus detection task paradigms do not consistently slow over the course of PAD (Körber et al., 2015a; Solís-Marcos et al., 2017). Furthermore, only one of the reviewed PAD studies compared multi-stimuli to manual driving (Solís-Marcos et al., 2017), so more research is needed in order to better characterize this approach to understanding vigilance decrements in PAD related to manual driving.

Detection tasks can also be implemented in more naturalistic ways. For example, Cisler et al. (2019) instructed drivers to detect whenever they see a certain billboard on a simulated highway. Response times slowed over a session of PAD; however, this change was not statistically significant. Other researchers have instructed participants to detect red cars in their simulated driving environment; again this did not show any significant change in response time throughout the drive (Heikoop et al., 2017). These results are inconsistent with results from single stimulus detection measures, however, both of these studies found evidence of vigilance decrements with other, non-detection task measures (Heikoop et al., 2017; Cisler et al., 2019).

Detection tasks are commonly used in driving research because they can be easily implemented in simulated and on-road driving scenarios. In general, the equipment is portable and simple to work with (e.g., attaching an LED to a windshield or placing a speaker in a car) and low cost. More naturalistic detection tasks leverage stimuli that are already in the driving environment so there is zero cost or equipment required. Furthermore, detection tasks provide data in the form of response times as well as accuracy, and we can monitor changes in these measures to see how vigilance decrements change over time.

One drawback of detections tasks is that some research suggests that the DRT may impose an additional cognitive load on the driver and impact driving performance (Stojmenova and Sodnik, 2018a, b). This increased cognitive load may stop drivers from becoming underloaded during PAD. Therefore, these tasks would not be able to monitor driver vigilance and may in fact help drivers to stay vigilant. If this were true, then it could help to explain why multi-stimulus detection task paradigms, which have significantly more stimuli than single stimulus detection task paradigms, do not show changes in performance during PAD (Körber et al., 2015a; Solís-Marcos et al., 2017). Even with single detection task paradigms, it is difficult to determine if a detection task is ideal for monitoring vigilance or is a cognitively demanding secondary task. Indeed, if a single stimulus detection task paradigm includes a frequent stimulus, then this could keep drivers engaged in the drive thus reducing the likelihood of observing vigilance decrements. Even at lower frequencies, if a detection task impacts driving performance, this would suggest that it is creating a cognitive state that is more similar to secondary task distracted driving, although others have argued that detection tasks place no additional demand on drivers and do not impact driving performance or vigilance (Strayer et al., 2019).

### Eye-Tracking

Another online measure used in PAD contexts to characterize vigilance decrements is eye-tracking, which can be done with either wearable or remote cameras (Merat et al., 2012, 2014; Körber et al., 2015a; Louw et al., 2015; Zeeb et al., 2015; Heikoop et al., 2017, 2018; Louw and Merat, 2017; Cisler et al., 2019; Gaspar and Carney, 2019; Jarosch et al., 2019). These cameras produce several metrics that have been attributed to vigilance changes in PAD. One common metric is whether or not drivers are looking at the forward roadway. This has clear implications for traffic safety because when drivers are not looking at the forward roadway, they may not attend to important events on-road. In one PAD study, Gaspar and Carney (2019) reported on a naturalistic experiment in which participants engaged in PAD conditions on an actual highway. They found that drivers spent less time looking at the forward roadway during PAD compared to manual driving (Gaspar and Carney, 2019), and this has been found in simulated PAD as well (Louw et al., 2015).

In addition to measuring where drivers are looking in PAD contexts, eye-tracking can also measure the percent of time the eyes are closed via an algorithm aptly named PERCLOS (Wierwille et al., 1994). This algorithm along with other analyses of driver blink frequency and duration have been associated with driver vigilance in PAD contexts. Specifically, during PAD, drivers showed increasing PERCLOS scores over the duration of the drive (Jarosch et al., 2019). More broadly, longer lasting and more frequent blinks have been associated with vigilance decrements in PAD (Merat et al., 2012; Heikoop et al., 2017).

Taken together, eye-tracking can be a useful online measure for vigilance in PAD contexts with clear implications for traffic safety. Another benefit to eye tracking is that is it can be implemented in a variety of different experimental settings. For example, dashboard mounted eye-trackers are particularly less of a hinderance because they can record head and eye movements without touching the driver. That said, eye-tracking in PAD contexts is not without some limitations. Eye-trackers are sensitive to changes in lighting conditions, which can change in dynamic real-world and simulated driving scenarios, making it difficult to distinguish signal from noise. This could be in part why some studies do not find differences in eye-tracking measures for PAD (Körber et al., 2015a), and it is not clear if this inconsistency outweighs the time and financial costs associated with using online eye-tracking measures in PAD contexts to characterize vigilance.

### Physiology

Different physiological measures have been used as online measures of driver vigilance in PAD. Electroencephalography (EEG) is one such metric that can produce two different types of data that have been associated with vigilance. Neural oscillations are rhythmic patterns of neural firing that we can interpret to understand patterns of brain activity in real-time. One such pattern that is commonly used to study attention consists of alpha frequency oscillations which range from 8 to 12 Hz. Alpha frequency oscillations increase in power, or magnitude squared, as attention processes decline and are also associated with lapses in attention to external stimuli (Klimesch, 2012; Borghini et al., 2014). In driving research, increased alpha power has been found during PAD as an online measure of vigilance decrements (Cisler et al., 2019).

In addition to measuring neural oscillations at different frequency bands with EEG, researchers can also average more acute neural responses to repeated stimuli and investigate event related potentials (ERP). One canonical ERP in vigilance research is the P300 in part because the amplitude is negatively associated with the amount of attention allocated to an infrequent or irregular task. Thus, reduced P300 amplitudes can be another indicator of vigilance decrements (Humphrey et al., 1994; Zhao et al., 2012). In one driving study, researchers measured ERPs in response to an oddball task performed while driving. P300 amplitudes to the detection task were significantly lower during PAD compared to manual driving, which was attributed to a vigilance decrement (Solís-Marcos et al., 2017).

Another type of physiological measure used as an online measure of vigilance is heart rate or HR. Outside of PAD research, HR measures have been commonly used to assess different types of cognitive demands while driving with increases in HR as cognitive load increases (Mehler et al., 2009). Within PAD research, decreased HR has often been associated with declines in vigilance. For example, Biondi et al. (2018) observed lower HR during PAD compared to manual driving on a real highway. In addition, drivers with lower HR respond more slowly to safety critical events (Funkhouser and Drews, 2016). Two PAD studies did not find significant differences in HR between PAD scenarios involving detection tasks and a no task control, but they did find that HR overall decreased over the course of the drive for all PAD scenarios (Heikoop et al., 2017, 2018).

Physiological measures are promising avenues for monitoring vigilance decrements in real-time. Collecting these measures may have little impact on driving performance since they do not require drivers to perform other tasks, and they have high temporal sensitivity. On the other hand, the equipment needed to record these data may be invasive or uncomfortable for participants to wear. EEG in particular requires drivers to minimize movements to reduce signal noise, which is difficult in driving settings since drivers need to move their heads to see the environment around them.

### Questionnaire Probes

One final online measure of vigilance decrements in PAD involves questionnaire probes. Questionnaires are traditionally used as offline measures; however, portions of questionnaires can be presented as online measures in the form of probes during the drive. Excerpts from the DSSQ, SSSQ, and think out loud protocols have been presented to drivers during PAD (Körber et al., 2015a; Heikoop et al., 2018; Cisler et al., 2019). The use of these probes can indicate a specific mental state of the driver at a specific point in time. For example, Körber et al. (2015a) probed drivers about mind-wandering after the first five minutes of driving and the last five minutes of the drive. A higher rate of mind-wandering was found near the end of a session of PAD which had induced a vigilance decrement (Körber et al., 2015a). Similarly, think-out-load protocols have also been used to monitor driver thoughts during PAD scenarios and have been associated with vigilance decrements (Heikoop et al., 2018).

Questionnaire probes, like pre/post questionnaires, are usually quick to implement and cost-effective, although they have some limitations to consider. Similar to concerns about detection tasks, questionnaire probes may impose a cognitive demand on drivers that can impact vigilance decrements. For example, if drivers know they will be asked to report how many times they have mind-wandered throughout the drive, then they may actively try to monitor their thoughts. Indeed, outside the realm of PAD research, the presence of a mind-wandering probe can impact how someone mind-wanders and the frequency of mind-wandering (Voss et al., 2018). Also, the presence of any type of questionnaire probe introduces another task into the driving environment and may redirect the driver’s attention away from the driving scenario or introduce another source of cognitive demand, which can be problematic for the online measurement of vigilance decrements.

## Discussion

More and more vehicles are being implemented with partially automated technologies despite the fact that PAD puts drivers in the precarious situation of having to monitor infrequent targets often in monotonous situations. This can lead to situations of cognitive underload in which drivers exhibit vigilance decrements. Before we can create countermeasures to these vigilance decrements, we must understand how vigilance decrements have been measured in PAD research. In this paper, we have reviewed commonly used offline and online measures of vigilance decrements during PAD, as well as some of their benefits and limitations. This is meant to provide researchers with an initial characterization on which future PAD research can build. We now turn to some unresolved issues based on the extant literature with the goal of highlighting additional areas for future research.

### Difficulty Discerning Causes of Vigilance Decrements in PAD

On the one hand, it is fairly clear that vigilance decrements in PAD are due to the fact that PAD requires drivers to monitor infrequent stimuli often during monotonous, highway driving. From decades of research on vigilance, as well as the articles specific to PAD reviewed in this paper, we can be confident there will be cognitive underload and subsequent vigilance decrements as auto manufacturers continue to produce vehicles capable of PAD. What is less clear is the underlying mechanism driving this underload.

From our characterization of this literature, it has become clear that there is a lack of clarity and consistency with regard to possible underlying causes of the vigilance decrements in PAD. Of the 20 articles reviewed, 11 mention mind-wandering, passive fatigue, or both as potential causes of vigilance decrements (see Table 2). For example, Biondi et al. (2018) mention how both mind-wandering and passive fatigue can come into play during vigilance decrements; along with other literature we have reviewed (Körber et al., 2015a, b; Louw et al., 2015; Cisler et al., 2019). Other articles only focus on how passive fatigue can lead to vigilance decrements (Saxby et al., 2008, 2013; Neubauer et al., 2012; Solís-Marcos et al., 2017; Jarosch et al., 2019). Louw and Merat (2017) is the only article that mentions mind-wandering and does not mention passive fatigue. In other words, if we rely solely on what researchers studying vigilance decrements in PAD say, then we are left with little consensus in terms of whether the observed characteristics of underload leading to vigilance decrements are driven by passive fatigue, mind-wandering, or both.

This lack of consistency may stem from the fact that the patterns of data attributed to one mechanism often overlap with patterns attributed to a different mechanism. In our review of offline measures, lower scores on the NASA-TLX have been attributed to vigilance decrements caused by passive fatigue (Saxby et al., 2008, 2013; Solís-Marcos et al., 2017), while non-PAD research has attributed lower NASA-TLX to mind-wandering (Zhang and Kumada, 2017; Neigel et al., 2019). Even the driver engagement questionnaires that have items explicitly mentioning mind-wandering have non-etheless been used in studies attributing vigilance decrements to passive fatigue, and have done so when used as both online and offline measures (Saxby et al., 2008, 2013; Neubauer et al., 2012; Körber et al., 2015a; Cisler et al., 2019). This also happens with several other online measures, for example, the slowing of responses measured by detection task paradigms in PAD has been characterized as vigilance decrements stemming from both mind-wandering and passive fatigue (Biondi et al., 2018). Safety critical events also show slowed responses during vigilance decrements in relation to passive fatigue (Saxby et al., 2008, 2013; Neubauer et al., 2012) and in one case to both passive fatigue and mind-wandering (Cisler et al., 2019).

When we look to other online measures, such as eye-tracking, we also find apparent inconsistencies. Indeed, eye patterns that have been associated with mind-wandering in non-PAD studies (Smilek et al., 2010; Uzzaman and Joordens, 2011; Krasich et al., 2018) are similar to the patterns associated with passive fatigue in both driving (Körber et al., 2015a; Jarosch et al., 2019) and non-driving studies (McIntire et al., 2014). For example, in a simulated air traffic control monitoring task, McIntire et al. (2014) found an increase in blink frequency and duration that was associated with vigilance decrements they attributed to passive fatigue. Within the realm of driving, Jarosch et al. (2019) found more blinks and longer blinks in PAD compared to driving while completing trivia quizzes, which they also attributed to passive fatigue. Furthermore, Körber et al. (2015a) found greater blink frequency and duration in passive fatigue conditions involving PAD. Interestingly, these eye patterns which they attributed to passive fatigue were also correlated with increased self-reported mind-wandering, and indeed, beyond PAD research, mind-wandering has been associated with similar eye-tracking patterns such as increased blinking (Smilek et al., 2010; Uzzaman and Joordens, 2011; Krasich et al., 2018).

One online measure in PAD that did differ in terms of patterns attributed to mind-wandering compared to patterns attributed to passive fatigue was HR. For example, HR patterns have been associated with mind-wandering in non-PAD studies with higher HR being reported as a marker for mind-wandering (Smallwood et al., 2004a, b, 2007; Ottaviani and Couyoumdjian, 2013). Yet in our review of vigilance decrements in PAD studies, often PAD scenarios were marked with lower HR (Funkhouser and Drews, 2016; Biondi et al., 2018). This could indicate that perhaps passive fatigue (rather than mind-wandering) was driving the vigilance decrements induced during these PAD studies. Future research is needed to replicate these specific findings.

Electroencephalography is another case than can further show patterns that have been related to both passive fatigue and to mind-wandering. Reduced P300 amplitudes are an indicator of declined vigilance in PAD literature (Solís-Marcos et al., 2017) and in non-PAD literature (Humphrey et al., 1994; Zhao et al., 2012). Baldwin et al. (2017) has attributed vigilance decrements shown by reduced P300 amplitudes to mind-wandering while driving. However, the ERP literature in our review related their results to passive fatigue (Solís-Marcos et al., 2017). Furthermore, Baldwin et al. (2017) found an increase in alpha frequency power in the same experiment; also attributing this to mind-wandering. In a PAD study, Cisler et al. (2019) found increased alpha power associated with vigilance decrements during PAD. The authors related these results to both mind-wandering and passive fatigue.

These inconsistencies in determining the cause of vigilance decrements during PAD are pervasive in the current literature. It is possible that passive fatigue and mind-wandering are competing theories that need further investigating. However, it is also possible that passive fatigue and mind-wandering are not mutually exclusive. In other words, it is possible that these two phenomena are more closely related than previously thought and can occur at the same time to contribute to vigilance decrements. This characterization may be more in line with that of Thomson et al. (2015) who proposed a resource-control account that combines both theories. This may be important to consider when evaluating the causes of vigilance decrements in future PAD research.

### Advocating for Multiple Measures

One potential avenue for moving forward is to use multiple measures to monitor vigilance decrements in PAD. The specific number and combination will most likely differ depending on the specific research question, as well as the equipment and other capabilities available to different research labs. Regardless, having at least more than a single measure can be beneficial for a number of reasons. For example, if researchers using EEG discovered that participant movement was particularly heightened in certain PAD contexts, this might prevent them from detecting a reliable signal from noise. If they also included offline measures, which are cheap and easy to implement, then perhaps not all would be lost. Not only is this redundancy beneficial in the event of data loss, but it also allows us to develop a better understanding of vigilance decrements by combining online and offline measures. Particularly because offline measures can provide information about how drivers were thinking and feeling during PAD, while online measures allow for a quantitative measure of vigilance decrements over time. Indeed, from the studies we reviewed, several used multiple measures perhaps in an attempt to maximize their chances of detecting possible vigilance decrements. For example, Neubauer et al. (2012) found similar workload scores for PAD and manual driving. Rather than conclude on this single measure that vigilance had not declined, they looked to additional measures and found slower responses to safety critical events during PAD compared to manual driving and lower post-task engagement scores after PAD, suggesting a vigilance decrement was present during PAD.

Across the studies we reviewed, 17 of the 20 articles presented data from multiple measures used to monitor vigilance decrements. However, only nine of these studies used a combination of online and offline measures. We encourage not only the use of multiple measures, but the use of both online and offline measures. This allows researchers to leverage the benefits of both types of measures. For example, the two offline measures we discussed have the benefit of being easy to use and cost effective, so it seems reasonable to include these in any future PAD research. The inclusion of online measures helps researchers to detect vigilance decrements in real-time, however, it does not make sense to use all online measures at once. For example, a combination of safety critical events, detection tasks, and questionnaire probes would require drivers to engage in multiple tasks and may impose an added cognitive demand which overloads drivers. Safety critical events are a direct way to monitor vigilance decrements, so as long as the research is conducted in a driving simulator they should be included. Detection tasks and questionnaire probes pose the risk of providing an added cognitive demand to the driver, so these measures are less ideal. However, if research is being done in the real-world then these two may be an acceptable option as long as all safety considerations are made. Eye-tracking devices can be used in any driving environment and are usually non-invasive to the driver, so we also recommend using eye-tracking measures whenever possible. Finally, driver physiology does not require drivers to perform any added tasks, so as long as a lab has the resources then one or multiple physiological measures should be included.

### Other Considerations for Measuring Vigilance Decrements in PAD

It can be difficult to distinguish between vigilance decrements due to underload and vigilance decrements due to sleepiness. We avoided articles that focused on sleepiness and PAD, however, the articles we did include are not immune to the effects of sleepiness. It is difficult to disentangle the effects of sleepiness and the effects of vigilance decrements due to underload, and thus it is possible that declines in task performance that appear to be due to underload are actually due to sleep related fatigue (Wierwille et al., 1994; Merat et al., 2012; Van Schie et al., 2012; Heikoop et al., 2017; Jarosch et al., 2019). Furthermore, eye patterns that can detect mind-wandering have been confounded with eye patterns that detect sleepiness (Stawarczyk et al., 2020). Sleepiness tends to increase throughout the drive, and this is not necessarily due to a particularly fatiguing drive (Jarosch et al., 2019). One simple way to account for sleep related fatigue is to use questionnaires that ask about sleepiness. For example, the Karolinska Sleep Scale (KSS) (Åkerstedt and Gillberg, 1990) has been presented to participants after a period of PAD (Solís-Marcos et al., 2017). Drivers have also been asked about sleepiness during the drive in the form of questionnaire probes (Jarosch et al., 2019). Monitoring driver sleepiness is not only important for understanding the current state of the driver, but it can also help us to better understand the results of other measures we use. For example, high PERCLOS scores may indicate a vigilance decrement due to underload but may also indicate high drowsiness (Wierwille et al., 1994; Merat et al., 2012; Heikoop et al., 2017; Jarosch et al., 2019). Therefore, we can use the KSS to clarify the result. If drivers report low sleepiness but high PERCLOS scores are observed, then we can more confidently attribute the PERCLOS patterns to underload rather than simply to sleepiness. This is another example of when having multiple measures in PAD can be beneficial.

Another important consideration when investigating vigilance decrements in PAD is the use of simulations. Driving simulations are low risk and can simulate a variety of driving environments, types of PAD, and seem to be preferred by the current research. Indeed, only two of the 20 articles we reviewed used data collected on-road. On-road research is higher risk and lacks experimental control, so it is not surprising that simulations are more commonly used. On the other hand, simulations may also be less arousing for the driver than real-world driving, so researchers must be aware of this when conducting future simulation research. Furthermore, simulations cannot create the same risks as real-world driving, so drivers may respond differently to safety critical situations than they would on the road (Caird and Horrey, 2011), though see McWilliams et al. (2018) for examples when performance in simulated driving corresponds to real-world driving.

A final concern about several measures we reviewed is their usability during real-world driving outside of research settings. It could be impractical to integrate some of these measures into consumer vehicles as part of the vehicles partial automation and safety systems. Offline measures require driver input that can only be provided after the fact, and therefore the data cannot be used to detect vigilance decrements in real-time, which is necessary if we hope to implement countermeasures to prevent vigilance decrements. Online measures may require cumbersome physiological sensors. For example, it is probably impractical for drivers to put on an EEG cap and electrodes whenever they get into their car. Therefore we need to continue the conversation between researchers and manufacturers to make effective and practical decisions. One measure that could be practically implemented in a real-world driving situation is eye-tracking. As mentioned, eye-tracking metrics are associated with vigilance decrements and are a promising tool to measure vigilance decrements in real-world settings. Eye-tracking may not be the only implementation, but researchers and manufacturers can build on the measures discussed so far to spark future innovations for PAD safety.

### Conclusion

Overall, PAD places drivers in a scenario where they are underloaded which can lead to vigilance decrements. Since drivers are expected to pay attention to the road at all times during PAD, vigilance decrements are not ideal because they can lead to dangerous situations on the road. In the future, researchers can explore countermeasures to these vigilance decrements, but first we must better understand how vigilance decrements are being measured in PAD. In this paper, we have reviewed commonly used offline and online measures of vigilance decrements in PAD, as well as some advantages and disadvantages of each measure. Combining multiple measures gives us the best chance of capturing vigilance decrements, and we hope this review can serve as part of the important dialogue needed between researchers and those developing and implementing newer forms of PAD with the goal of providing countermeasures to vigilance decrements in PAD.

## Author Contributions

TM and NW were both involved in conceptualization, writing, and editing this manuscript. Both authors contributed to the article and approved the submitted version.

## Conflict of Interest

The authors declare that the research was conducted in the absence of any commercial or financial relationships that could be construed as a potential conflict of interest.
